# The human plasma anellome exhibits age- and sex-dependent patterns with links to cardiometabolic health in older adults

**DOI:** 10.3389/fmicb.2025.1716110

**Published:** 2026-01-15

**Authors:** Yakhouba Kane, Yingying Ma, Beibei Yan, Xiuli Zhao, Ting Ge, Yanpeng Li, Le Cao, Min Zhang, Zhenzhou Wan, Ting Zhang, Chiyu Zhang

**Affiliations:** 1 Shanghai Public Health Clinical Center, Fudan University, Shanghai, China; 2 Medical Laboratory, Taizhou Fourth People’s Hospital, Taizhou, Jiangsu, China; 3 Gut Microbiota and Metabolic Research Center, Institute of Pediatric Infection, Immunity and Critical Care Medicine, School of Medicine, Shanghai Jiao Tong University, Shanghai, China

**Keywords:** plasma virome, metagenomics, anelloviruses, cardiometabolic diseases, cytokines, HERV-K

## Abstract

The human plasma virome is dominated by anelloviruses which are increasingly associated with several clinical conditions including among others HIV-1, COVID-19, autoimmune diseases, and cardiovascular and metabolic diseases. Due to their high genetic divergence, most studies investigated human anellome at broad family or genus level. These approaches obscure the contributions of specific anellovirus species to clinical conditions. We conducted plasma metagenomics in 218 individuals from young (0–16 years) and old (63–100 years) cohorts to resolve the anellome at the species level and examine its patterns across age, sex, and associations with cytokines and cardiometabolic outcomes. Older adults exhibited near-universal anellovirus detection and significantly higher abundance compared with youth. Species-specific analysis revealed that *Alphatorquevirus_homin1 and Alphatorquevirus_homin13* were markedly enriched in diseased older adults. Predictive modeling based on machine learning algorithms distinguished disease status in the young cohort with high accuracy (AUC = 0.86), but performance was limited in the elderly (AUC = 0.58), suggesting a lack of diagnostic value in advanced age. Specific species abundances and diversity were associated with stroke and coronary heart disease, while cytokine correlations revealed module-specific immune signatures: Gammatorquevirus-dominated modules associated positively with pro-inflammatory cytokines and growth factors (e.g., IL-1β, IL-15, VEGF), whereas Beta- and some Alphatorquevirus-dominated modules showed predominantly negative correlations with several inflammatory and regulatory mediators (e.g., IL-6, TNF-*α*, IL-10). These findings demonstrate that the anellome is influenced by age and immune status and shows associations with cardiometabolic health, although these relationships do not guarentee diagnostic or causal significance. Additonally, we found no significant differences of Human endogenous retrovirus K Env expression between disease and healthy controls. This work underscores the importance of resolving human anollome to species level in future longitudinal studies to strengthen their clinical significance and biomarker potential.

## Introduction

The human blood virome is currently recognized as a structured and biologically meaningful component of the human holobiont rather than a mere collection of incidental nucleic acids (Nayfach et al., 2021b). Systematic metagenomic analyses have cataloged extensive viral diversity across tissues, with Anelloviridae representing the most prevalent constituents of the plasma DNA virome (Kumata et al., 2020; Arze et al., 2021; Kaczorowska et al., 2023; Pyöriä et al., 2024). This viral family has high prevalence, genetic diversity, and chronic persistence in humans without clear clinical significance (Kaczorowska et al., 2023; Modha et al., 2025; Sasa et al., 2025).

Recent studies have illuminated complex relationships between blood virome components and human diseases (Blatter et al., 2020; Li et al., 2022; Cao et al., 2023; Sabbaghian et al., 2024; Sasa et al., 2025). Integration and reactivation of herpesviruses including human herpesvirus 6 are associated with severe outcomes in immunocompromised individuals and autoimmune conditions (Mihalić et al., 2024; Pyöriä et al., 2024; Sasa et al., 2025). Moreover, elevated anellome or its composition has been linked to subclinical graft rejection, post-acute COVID-19 sequelae, HIV/HBV/HCV coinfection outcomes in intravenous drug users, and autoimmune diseases (Thijssen et al., 2020; Li et al., 2022; Maguire et al., 2024; Ma et al., 2025; Sasa et al., 2025). In addition, human endogenous retrovirus K (HERV-K), particularly the HML-2 subgroup, can be transcriptionally reactivated in cancer, autoimmune diseases, and during exogenous viral infections (Subramanian et al., 2011; Kitsou et al., 2023; Bao et al., 2024). This reactivation can drive expression of envelope proteins and virus-like particles, stimulating innate immune pathways and further linking endogenous retroelements to virome-host immune crosstalk.

Viral infections and persistent nucleic acids drive inflammation, endothelial dysfunction, and immune dysregulation, processes directly linked to several cardiometabolic conditions (Thijssen et al., 2023; Clarke et al., 2024; Miller et al., 2024; Boparai et al., 2025; Nguyen et al., 2025; Sasa et al., 2025; Zhou and Feng, 2025). Recent studies implicated acute infections, such as those caused by influenza virus, severe acute respiratory syndrome coronavirus 2 (SARS-CoV-2), varicella-zoster virus (VZV), and cytomegalovirus (CMV), in elevate short-term risks of ischemic stroke and/or acute myocardial infarction (AMI), while evidence linked SARS-CoV-2 to new-onset hypertension and diabetes (Clarke et al., 2024; Miller et al., 2024; Boparai et al., 2025; Nguyen et al., 2025; Zhou and Feng, 2025). Persistent blood-borne viruses, particularly anelloviruses, were also associated in chronic immune perturbation that co-occurs with cardiometabolic disease (Thijssen et al., 2023; Sasa et al., 2025). Notably, a study found that high Torque Teno viruses (TTV) viremia independently increased ischemic heart disease (IHD) risk and correlated with high pro-inflammatory cytokines and immunosenescence markers, suggesting potential mechanistic link between TTV, systemic inflammation, and IHD risk (Giacconi et al., 2024). However, TTV qPCR targets multiple *Alphatorquevirus* species and cannot pinpoint the species or lineages that drive the association, and the roles of other anellovirus genera in cardiovascular events are yet to be explored.

Age is a major determinant of blood virome dynamics. Progressive immune remodeling through immunosenescence and inflammaging weakens antiviral defense and promotes chronic low-grade inflammation, creating a permissive environment for viral persistence and reactivation (Liu et al., 2023; Gadoth et al., 2024; Sabbaghian et al., 2024). As a result, the composition of the circulating virome is likely to both mirror and influence age-related patterns of immune dysregulation and disease risk.

Despite these advances, major gaps remain. Studies linking viral infections to increased risk of cardiovascular or metabolic disease are scarce. Notably, most human anellome studies focused on well-characterized anellovirus genera (*Alpha*-, *Beta*-, and *Gammatorquevirus*) or relied on qPCR-based quantification of TTV, approaches that obscure within-family genetic diversity and species-specific signals (Giacconi et al., 2024; Sasa et al., 2025). This leaves the relationships between anelloviruses and host immune status poorly defined. To address this, we conducted both genus and species-resolution analyses of the plasma virome, alongside measurements of cytokines and HERV-K envelop expression, in two age-stratified cohorts comprising healthy controls and individuals with chronic or age-related conditions. This study aims to characterize the plasma virome, with a particular focus on human anellome prevalence, abundance, diversity, and to identify relationships between anellovirus species, immune markers, and HERV-K Env expressions.

## Materials and methods

### Study subjects and ethical statement

**Figure 1:**
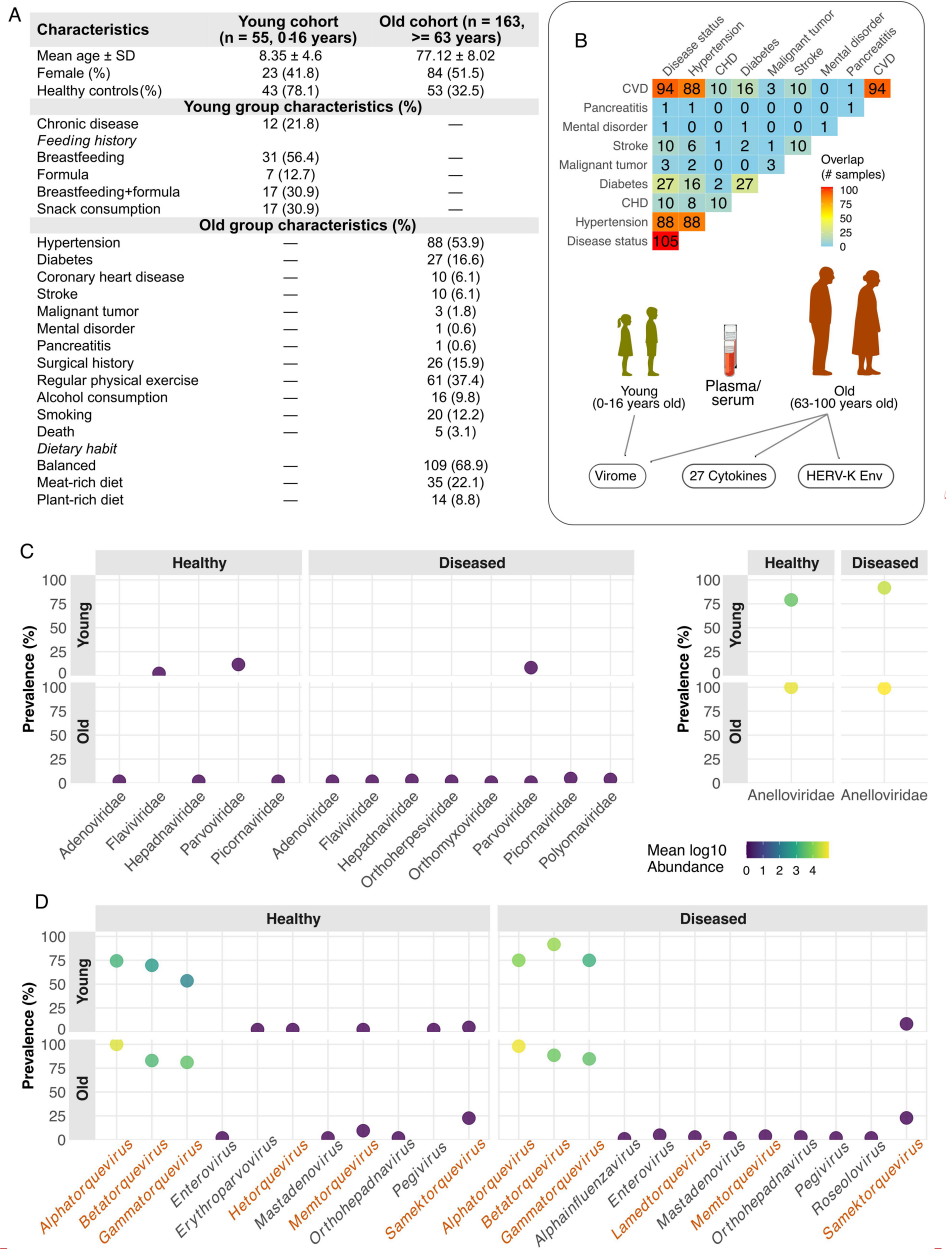
Study cohorts and overview of plasma virome. **(A)** Description of the study population consisting of two age cohorts including young and old adult cohorts along with disease and lifestyle characteristics. **(B)** The number of samples shared across age-related commorbities (old cohort) and the types of data generated in this study: the plasma virome, 27 human cytokines, and the expresssion levels of HERV-K envelop protein. Cytokine and HERV-K measurements were performed only for only the old cohort individuals. The arrows indicate the type of sample collected. **(C,D)** Prevalence and abundance (mean log10) of plasma viruses at the family **(C)** and genus **(D)** levels. In **(D)**, anellovirus genera are shown in red and other eukaryotic viral genera in black.

This study investigated the plasma virome and immune markers between young (0–16 years old) and old (63–100 years) cohorts associated with chronic (young) and age-related (old) diseases (Figure 1A). Chronic conditions were defined as immune thrombocytopenia, adenoid hypertrophy, allergic rhinitis, sinusitis, asthma, or Crohn’s disease. The study was approved by both the Ethics Committee of Taizhou Fourth People’s Hospital (approval number: 2023-EC/TZFH-015) and the Regional Ethics Committee of Shanghai Children’s Hospital (approval number: 2025R081-E02). Written informed consent was obtained from all participants or their parents (for minors) before enrollment and sample collection. Blood samples of the old age cohort were collected from participants recruited at Taizhou Fourth People’s Hospital (Jiangsu Province, China) June and November 2023, while those of young age cohort, blood samples were collected from minor subjects recruited at Shanghai Children’s Hospital between August 2024 to November 2024. Both plasma and serum fractions were obtained, with serum specifically used to quantify the expression levels of HERV-K envelope protein.

### Plasma virome

#### Sample processing and next-generation sequencing

The overview of the study workflow is shown in Supplementary Figure S1A. A negative control consisting of nuclease-free water was added to each sample batch to remove potential contaminants. Plasma samples were centrifuged 12,000 rpm for 10 min at 4 °C to remove cellular debris. The supernatant was treated with an enzyme cocktail (DNase I, RNase A, Benzonase) at 37 °C for 1.5 h to digest free-ranging nucleic acids. Total DNA/RNA was extracted from 300 μL of the treated plasma using Qiagen MiniElute Viral Spin Kit (Qiagen) and reverse-transcribed and amplified with REPLI-g WTA Single Cell Kit (Qiagen). The amplification products were purified with Monarch PCR & DNA Cleanup Kit (New England Biolabs) and quantified using the Qubit dsDNA Assay Kit (Life Technologies, CA, USA). Metagenomic libraries were prepared with the NEBNext Ultra DNA Library Prep Kit for Illumina (NEB, USA). Library quality and size distribution were assessed via Agilent 2,100 Bioanalyzer and qualified libraries were sequenced on Illumina NovaSeq platform (Illumina, USA) to generate 5G of 2 × 150 bp paired-end reads per sample.

### Virome data analysis

The workflow for virome analysis is highlighted in Supplementary Figure S1B. Raw reads were processed with fastp v0.23.3 for adapter trimming, low complexity filtering, and minimum length of 30 nt. FastQC was used for quality control (Babraham Bioinformatics—FastQC A Quality Control tool for High Throughput Sequence Data, 2025). Cleaned reads were aligned to the human genome HG38 using Bowtie2 v2.4.5 (−-sensitive local, −-no-unal), and host subtracted reads were *de novo* assembled using MEGAHIT v1.2.9 with a minimum contig length of 300 bp (Langmead and Salzberg, 2012; Li et al., 2015). The quality and completeness of viral contigs were assessed using CheckV v1.0.1 and contigs with at least one viral gene were extracted using Sekit v2.4.0 (Nayfach et al., 2021a). The suspected viral contigs were taxonomically annotated using both BLASTN and DIAMOND BLASTX using NCBI NT and NR databases (Altschul et al., 1990; Buchfink et al., 2015). A custom python script was used to filter contigs using a decision matrix. First, both blast outputs were merged by contig ID and contigs were further filtered as follows: (1) ≥ 70% identity, ≤1 × 10^−10^ e value, and ≥100 bp alignment for BLASTN; (2) ≥ 30% identity, ≤1 × 10^−5^ e value, and ≥ 50 aa for DIAMOND BLASTX. Contigs passing both filters were subjected to a manual assessment for inclusion. Lineage details of the final viral contigs were obtained using Taxonkit 0.14.2(Shen and Ren, 2021). Raw reads were mapped back to retained viral contigs using bbmap and alignment stats calculated with Samtools v1.10 (Li et al., 2009). To reduce technical noise and focus on biologically plausible bloodborne signals, all downstream analyses were restricted to eukaryotic viruses.

### Anellovirus analysis

ORF1 coding sequences were retrieved from the SCANellome anellovirus database, as described in Laubscher et al. (2023), which encompasses the complete set of anellovirus ORF1 sequences available in NCBI GenBank^1^ (released March 31, 2023). These sequences were used to construct a custom BLASTN database. Protein accession numbers of anellovirus contigs were batch-queried via NCBI Entrez Direct to identify putative ORF1 regions. Contigs ≥1,000 bp in length with valid ORF1 were retained and aligned against the SCANellome database to assign genus- and species-level classifications. An abundance table was generated by mapping quality-filtered reads back to the classified anellovirus contigs.

### Cytokines screening

Plasma concentrations of 27 cytokines and chemokines (IL-1β, IL-1rα, IL-2, IL-4, IL-5, IL-6, IL-7, IL-8, IL-9, IL-10, IL-12, IL-13, IL-15, IL-17, Eotaxin, FGF-basic, G-CSF, GM-CSF, IFN-*γ*, IP-10, MCP-1, MIP-1*α*, PDGF-BB, MIP-1β, RANTES, TNF-α, and VEGF) were measured using the Bio-Plex Pro Assay Kit (Bio-Rad) according to the manufacturer’s protocol. Assays were performed in duplicate, and mean values were used for analysis. Cytokine concentrations were determined using a Bio-Plex 200 system with standard curves generated from known standards.

### HERV-K ELISA assay

Enzyme-Linked Immunosorbent Assay (ELISA) was used to quantify the HERV-K envelope protein in the serum samples using the Human Endogenous Retrovirus Envelope HERV-K (HERVKenV) ELISA kit (Aifang Biotechnology, China). Briefly, serum samples were diluted according to the kit manufacturer’s recommendations and added to a 96-well plate pre-coated with capture antibodies. After incubation, a detection antibody was further added and the bound proteins were quantified using an Infinite F50 microplate reader at an optical density of 450 nm. Each sample was analyzed in triplicate to ensure accuracy.

### Statistical analysis

All analyses were performed in R v4.5.1 (R: The R Project for Statistical Computing, 2025). We first defined cardiovascular disease (CVD) for individuals with a recorded history of stroke, coronary heart disease (CHD), and/or hypertension. The term cardiometabolic disease was used to indicate the presence of diabetes and at least one composite CVD. For univariate comparisons of abundance, viral features were log10-transformed after the addition of a pseudocount (half the minimum non-zero value for each feature) (Wei et al., 2018). For multivariate and correlation analyses, we addressed the challenge of zeros using multiplicative replacement and subsequently transformed the data to centered log-ratio (CLR) coordinates to account for the closed nature of relative abundance data (Martín-Fernández et al., 2003).

To assess associations between individual viral species and clinical outcomes, we modeled species presence against each binary disease endpoint. We first compared case and control prevalence using Fisher’s exact test. For species occurring in at least 10% of samples, associations were evaluated by standard logistic regression adjusted for age, sex, smoking, alcohol use, physical activity, and dietary habit. For species with 5–10% prevalence or those showing separation, Firth’s (1993)bias-reduced logistic regression was applied to ensure stable estimates. Species below 5% prevalence were excluded from modeling. Variance inflation factors were examined to confirm that covariates did not introduce multicollinearity, and covariates were only limited to age and sex if convergence failed. Odds ratios, *p*-values, and false discovery rate (FDR)-adjusted p-values were extracted for each species-disease pair and summarized for visualization (Benjamini and Hochberg, 1995).

To evaluate the multivariate predictive power of the virome, we trained supervised learning models to classify disease status based on the top 30 prevalent anellovirus species, prioritized by prevalence and univariate effect size. Species abundances were binarized (presence/absence), and the data were partitioned into a training set (70%) and a held-out test set (30%). We implemented four distinct classifiers including random forest (RF), logistic regression (glm), support vector machine with a radial basis kernel (svm), and extreme gradient boosting (XGBoost) with hyperparameters optimized through 5-fold cross-validation to maximize the area under the receiver operating characteristic curve (AUC) (McCullagh, 1984; Cortes and Vapnik, 1995; Breiman, 2001; Chen and Guestrin, 2016). Final model performance was evaluated by the AUC on the independent test set. We extracted variable importance metrics from each model to identify the most contributory viral features. This analysis was repeated separately in each cohort to identify age-specific viral signatures that may serve as candidate biomarkers.

Alpha diversity metrics (Shannon, richness, Simpson) were calculated at the viral family level and for Anelloviridae at the genus and species levels using vegan (R package v. 2.7) (Oksanen et al., 2025). Group comparisons were assessed using non-parametric tests (Wilcoxon rank-sum and Kruskal-Wallis), with Dunn’s test for post-hoc pairwise comparisons. Also, we reported non-parametric effect sizes, including median differences and Cliff’s delta where appropriated.

Circulating cytokines and HERV-K were log-transformed and viral abundances were CLR-transformed; all values were residualized for age, sex, smoking, alcohol use and physical activity prior to downstream analysis. Spearman correlations were computed between residualized virus and cytokine values. Exploratory associations were considered at rho ≥ 0.30. For visualization and focused reporting, we retained correlations with absolute rho ≥ 0.40 and sample size ≥ 10. Multiple testing correction using the Benjamini–Hochberg procedure was applied within each hypothesis family. Median correlations were summarized into a single virus × cytokine/HERV-K matrix by taking the median Spearman rho across disease contexts. Viruses were grouped by hierarchical clustering on the median-correlation matrix and the tree was cut into modules (Langfelder and Horvath, 2008). For species-level inspection, top viruses with the most retained cytokine associations were displayed as disease-stratified facet panels.

## Results

### Study cohort and overview of plasma virome

We analyzed 218 metagenomes from two age-stratified cohorts (Figures 1A,B, Supplementary Table S1). The young cohort included 55 children, mean age 8.3 ± 4.7 years; 23 females (41.8%), and 12 had chronic conditions (21.8%). Feeding history was breast milk 31 (56.4%), formula 7 (12.7%) and mixed 17 (30.9%); 17 (30.9%) reported frequent snacking. The older cohort comprised 163 adults, mean age 77.1 ± 8.0 years; 84 females (51.5%). Common comorbidities were hypertension 88 (54.0%), diabetes 27 (16.6%), coronary heart disease (CHD) 10 (6.1%), stroke 10 (6.1%), malignant tumor 3 (1.8%), mental disorder 1 (0.6%), pancreatitis 1 (0.6%). Lifestyle factors included physical exercise 26 (16.0%), alcohol use 16 (9.8%) and smoking 20 (12.3%). Diet was categorized as balanced in 109 (69.0%), meat-enriched in 35 (22.2%) and vegetable-enriched in 14 (8.9%).

Viral mining resulted in 7,107,848 contigs of which 147,095 showed potential viral hits. CheckV quality assessment revealed 1,651 complete, 4,879 high-quality, and 4,954 medium-quality viral contigs (Supplementary Table S2). Both metagenomic contigs and total viral hits were enriched in aged individuals (Supplementary Figures S2A,B). In young cohort, viral contigs showed a dip in age bin 5–10 years followed by gradual increase (Supplementary Figures S2C,D); and complete viral contigs were significantly higher in young diseased participants (Supplementary Figure S2E).

The plasma virome was dominated by Anelloviridae (Figure 1C), prevalent in both young (91.7% diseased, 79.1% healthy) and old (99.0% diseased, 100% healthy) cohorts. Anelloviridae showed the highest mean log10 abundance, substantially greater in the old cohort (diseased: 4.85 ± 0.90, healthy: 4.81 ± 1.02) than the young (diseased: 4.19 ± 1.64, healthy: 3.09 ± 1.86). Other eukaryotic viral families were sporadically detected (<12%) at markedly lower abundances (mean log10 < 0.35). This signal was driven by three well-characterized anellovirus genera with *Alphatorquevirus* being near-universal in older adults (98.7%) with high abundance (mean log10 4.65 ± 0.97) but was less common (74.5%) and abundant (2.75 ± 1.84) in the young (Figure 1D). *Betatorquevirus* and *Gammatorquevirus* were also highly prevalent (86.7, 83.5%) and abundant (mean log10 ~ 3.38, 3.25) in the old cohort, with reduced levels in the young. Notably, *Samektorquevirus* a fourth anellovirus genus was detected with a prevalence of 22.8%. All other anellovirus genera were detected at very low prevalence (<5% overall) and were not analyzed further. In contrast, other eukaryotic viral families were sporadically detected. *Erythroparvovirus* (Parvoviridae) signals were most apparent in children (healthy: 11.6% prevalence, mean log10 ~ 0.34), while *Enterovirus* (Picornaviridae) (4.8%, ~0.15) and *Alphapolyomavirus* (Polyomaviridae) (3.8%, ~0.11) had modest detections in the older diseased group. All other remaining genera were rare (<3%).

### Dominant anellovirus species showed age and disease-status patterns

**Figure 2:**
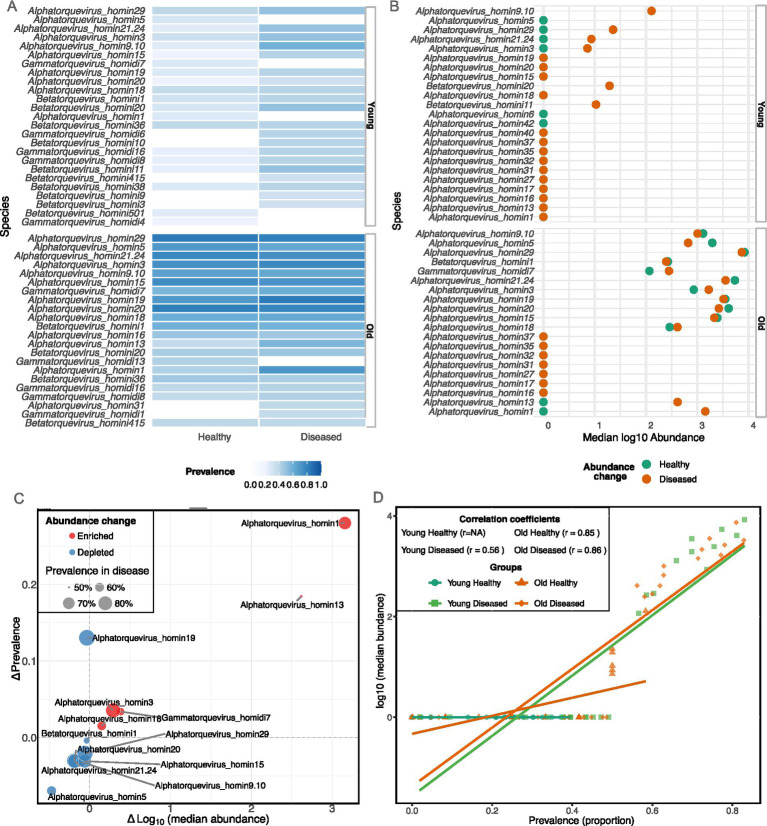
Disease status and cohort-associated alterations in the top anellovirus species. **(A,B)** The top 20 species by prevalence and abundance highlighting differences between cohorts and disease status. **(C)** Disease-associated shifts in anellovirus species within the older adult cohort. The change (*Δ*) in median log_10_ abundance (diseased vs. healthy) is plotted against the change in prevalence (Δ percentage). Point size corresponds to the species’ prevalence in diseased individuals, highlighting the most clinically relevant agents. **(D)** Prevalence-abundance relationship of the top 20 anellovirus species. Point size reflects overall median abundance, illustrating a positive correlation between a species’ detection frequency and its typical abundance level. NA, no variance.

In total, 202 anellovirus species were detected, including 22 *Alphatorquevirus*, 111 *Betatorquevirus*, 59 *Gammatorquevirus*, 5 *Samektorquevirus*, 2 *Memtorquevirus*, and one species from each of the remaining genera, with *Alphatorquevirus* species dominating across cohorts (Figures 2A–D). In older diseased individuals, the most prevalent were *Alphatorquevirus_homin19* (82.9%; median log10 3.51), *Alphatorquevirus_homin29* (80.95%; 3.87), *Alphatorquevirus_homin20* (78.10%, 3.42), *Alphatorquevirus_homin3* (77.14%, 3.22) and *Alphatorquevirus_homin1* (71.43%, 3.16). The older healthy subjects shared this core profile but was also characterized by a strong presence of *Alphatorquevirus_homin21.24* and *Alphatorquevirus_homin15* at abundances comparable to the top species (Figures 2A,B). Species with larger median log10 abundance (diseased vs. healthy) included *Alphatorquevirus_homin1* (3.16 vs. 0.00), *Alphatorquevirus_homin13* (2.62 vs. 0.00), *Alphatorquevirus_homin3* (3.22 vs. 2.93) and *Gammatorquevirus_homidi7* (2.45 vs. 2.06) (Figure 2B). In contrast, commonly detected species such as *Alphatorquevirus_homin29* (3.87 vs. 3.93) and *Alphatorquevirus_homin20* (3.42 vs. 3.61) showed limited differences.

In young, diseased samples, the virome composition was distinct. In young diseased group, the most notable species was *Alphatorquevirus_homin9.10*, which had the highest prevalence (58.3%) and abundance (median log10 2.11) in this cohort (Figures 2A,B). Other prominent species in young diseased individuals included *Alphatorquevirus_homin29* (50.0%; 1.36) and *Betatorquevirus_homini20* (50.0%; 1.29). In contrast, the top species in young healthy subjects, such as *Alphatorquevirus_homin29* (39.5%; 0.0) and *Alphatorquevirus_homin3* (34.9%; 0.00) had prevalence below 50%. Across cohorts, several *Betatorquevirus* and *Gammatorquevirus* species are present among the top-ranked species but with lower median abundances than dominant *Alphatorquevirus* species; for example, *Betatorquevirus_homini1* (old diseased 58.1%; 2.39), *Gammatorquevirus_homidi7* (old diseased prevalence 60.0%; 2.45), while in young healthy these taxa had generally lower prevalence and abundance.

The strongest disease-associated virome changes were observed in two anellovirus species: *Alphatorquevirus_homin1* (Δprevalence = +28.0%; Δmedian log10 abundance = +3.16) and *Alphatorquevirus_homin13* (+18.5%; +2.62), as shown in Figure 2C. A secondary cluster exhibited modest positive associations: *Alphatorquevirus_homin19* (+13.0%; −0.03), *Alphatorquevirus_homin3* (+3.6%; +0.29), *Gammatorquevirus_homidi7* (+3.4%; +0.38), and *Alphatorquevirus_homin18* (+1.5%; +0.15). The largest negative change was seen for *Alphatorquevirus_homin5* (−6.95%; −0.47), with smaller reductions (~ − 3–4%, −0.11 to −0.19) in several other Alphatorquevirus species. Notably, in older individuals, the most prevalent anellovirus species were also the most abundant, a pattern not observed in the young cohort (Figure 2D).

### Anellovirus genera show stroke-associated enrichment and CHD-specific depletion

**Figure 3:**
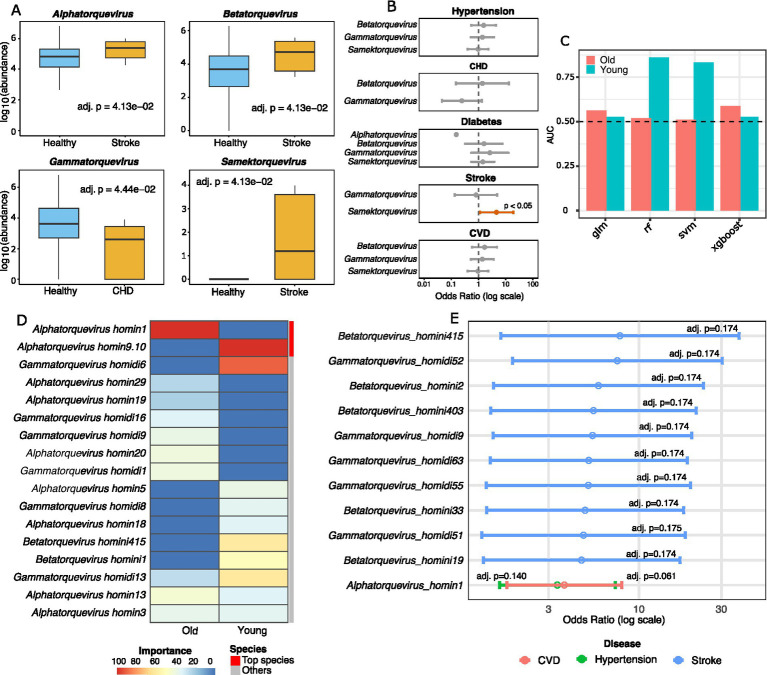
Association analysis of anelloviruses in age-related diseases and predictive modelling. **(A)** Alterations in anellovirus genus abundance between patients with specific diseases and healthy controls. Only genera with significant differences (adjusted *p* < 0.05) are displayed. Stroke: *Alphatorquevirus, Betatorquevirus, Samektorquevirus*; Coronary heart disease (CHD): *Gammatorquevirus*. **(B)** Association between the prevalence of anellovirus genera and specific diseases, analyzed by logistic regression. Genera with a statistically significant odds ratio (*p* < 0.05) for a given disease are highlighted in red. **(C)** Discriminative performance of four supervised learning models: Random Forest (RF), logistic regression (GLM), support vector machine with a radial basis kernel (SVM), extreme gradient boosting (XGBoost) trained on viral species features to predict disease status in young and older cohorts. **(D)** Clustered heatmap of variable importance scores for the top predictive viral species, derived from the best-performing model in each cohort [XGBoost for the older cohort and Random Forest for the younger cohort, as shown in **(C)**]. **(E)** Logistic regression of anellovirus species prevalence across disease types. Error bars represent 95% confidence intervals.

At the genus level, disease status was associated with significant alterations in viral abundance (Figure 3A). Stroke patients exhibited enrichment of *Alphatorquevirus*, *Betatorquevirus*, and *Samektorquevirus* (adj. *p* < 0.05), while *Gammatorquevirus* was significantly depleted in CHD (adj. *p* = 0.0444). Logistic regression confirmed a specific positive association between the presence of *Samektorquevirus* and stroke (OR = 4.62; 95% CI: 1.12–19.05; *p* = 0.034) (Figure 3B). No other genera were significantly associated with CHD, diabetes, hypertension, or the composite CVD status.

### Species-level anellovirus profiles predict disease status across age groups

Supervised classification models revealed stark cohort differences (Figure 3C). In the young cohort, random forest achieved the highest predictive power for disease status (AUC = 0.861), while all models performed poorly in older cohort (AUC range: 0.511–0.588), with XGBoost performing best. Variable importance analysis identified distinct predictive species (Figure 3D). In young cohort, *Alphatorquevirus_homin9.10* and *Alphatorquevirus_homin18* were consistently top-ranked across multiple models, while, *Alphatorquevirus_homin13* was a key predictor in rf and glm models. In the old cohort, *Alphatorquevirus_homin1* dominated, followed by *Alphatorquevirus_homin13*, and *Alphatorquevirus_homin20* (Figure 3D, Supplementary Table S3). Notably, specific anellovirus species were associated with cardiovascular outcomes in the older cohort (Figure 3E). *Alphatorquevirus_homin1* demonstrated stronger dose-responsive trends with increased odds for composite CVD (OR = 3.7, 95% CI: 1.72–7.96; adjusted *p* = 0.061) and hypertension (OR = 3.37, 95% CI: 1.56–7.28; adjusted *p* = 0.14). Furthermore, multiple *Gammatorquevirus* and *Betatorquevirus* species displayed a limited association with moderate increased odds for stroke.

### Disease and lifestyle shape anellovirus diversity in aged adults

**Figure 4:**
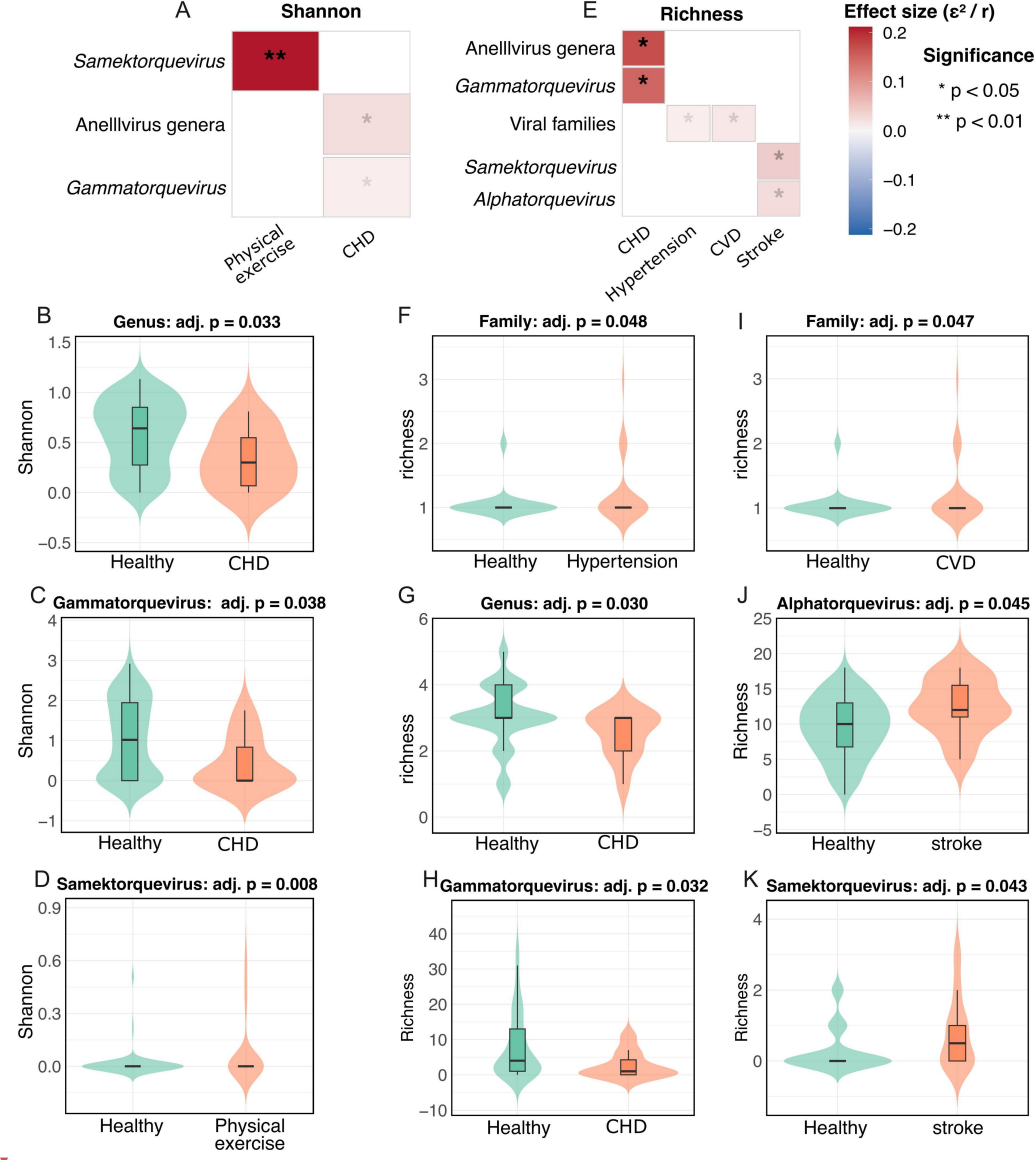
Associations between anellovirus alpha diversity and clinical traits in the older cohort. **(A)** Heatmap of effect sizes (ε^2^ for Kruskal-Wallis, *r* for Wilcoxon) from Shannon diversity analyses across all tested disease states and lifestyle factors. **(B)** Boxplots illustrating pairwise comparisons of Shannon diversity for significant associations identified: anellovirus genus-level diversity in coronary heart disease (CHD) versus healthy controls. **(C)** *Gammatorquevirus* species-level diversity in coronary heart disease (CHD) versus controls. **(D)** *Samektorquevirus* species-level diversity in individuals grouped by physical exercise level. **(E)** Heatmap of effect sizes for species and genus richness across all tested conditions. **(F)** Boxplots of richness for specific significant associations identified: viral family-level richness in hypertension. **(G)** Anellovirus genus-level richness in CHD. **(H)** *Gammatorquevirus* species richness in CHD. **(I)** Viral family-level richness in CVD. **(J)** *Alphatorquevirus* species richness in stroke. **(K)** *Samektorquevirus* species richness in stroke.

Analysis of alpha diversity revealed significant associations between anellovirus community structure and host traits in the older cohort (Figures 4A–K). Shannon diversity was altered across several conditions (Figures 4A–D). Notably, *Samektorquevirus* species diversity higher in physically active individuals (Figure 4D), whereas overall anellovirus genus diversity and *Gammatorquevirus* species diversity were reduced in the context of CHD, respectively (Figures 4B,C).

Species richness also varied markedly across cardiovascular conditions (Figures 4E–K). Viral family richness was marginally elevated in individuals with hypertension and composite CVD (Figures 4F,I), while richness of anellovirus genera decreased in CHD and for Gammatorquevirus species in CHD (Figures 4G,H). In contrast, species richness was significantly increased for *Alphatorquevirus* and *Samektorquevirus* in stroke patients (Figures 4J,K).

### Anellovirus prevalence is male-biased with modest lifestyle associations

Demographic analysis revealed strong sex-dependent patterns in anellovirus prevalence, with 33 species showing significantly higher odds in males (adj.*p* < 0.05) (Supplementary Table S4). The most pronounced male biases were observed in *Gammatorquevirus* and *Betatorquevirus* genera, particularly *Gammatorquevirus_homidi9* (OR = 10.05) and *Betatorquevirus_homini10* (OR = 9.29). While smoking was moderately linked with reduced prevalence of several species, physical exercise showed trends toward increased prevalence for multiple *Alphatorquevirus* species, particularly *Alphatorquevirus_homin21.24* (OR = 3.22), but these were not statistically significant after FDR adjustment. Other lifestyle factors showed no associations.

### Older males show higher alpha- and Gammatorquevirus diversity in healthy context

In healthy individuals, anellovirus community structure displayed age- and sex-specific patterns (Supplementary Figure S3). Simpson index at viral family level was higher in younger adults (Simpson index adj. *p* = 0.002), while male sex was moderately associated with higher anellovirus genus richness (Supplementary Figures S3A,B). At the genus level, *Alphatorquevirus* species showed the strongest age-associated differences, with both Shannon diversity (adj. *p* = 6.08 × 10^−5^) and richness (adj. *p* = 9.73 × 10^−5^) in the older cohort (Supplementary Figures S3C,D). Among the older adults, males showed further enrichment in *Alphatorquevirus* species (Shannon adj. *p* = 0.0419; richness adj. *p* = 0.0251) (Supplementary Figures S3E,F), along with a modest male-associated increase *Gammatorquevirus* species, while *Samektorquevirus* diversity was relatively higher in older females (adj. p ~ 0.057) (Supplementary Figures S3G–I). Although some effects were borderline significance after adjustment, their consistent direction across multiple metrics suggests reproducible age- and sex-specific structuring of anellovirus communities in the absence of overt disease.

### IL-1r*α* and G-CSF distinguish disease and cardiovascular states

**Figure 5:**
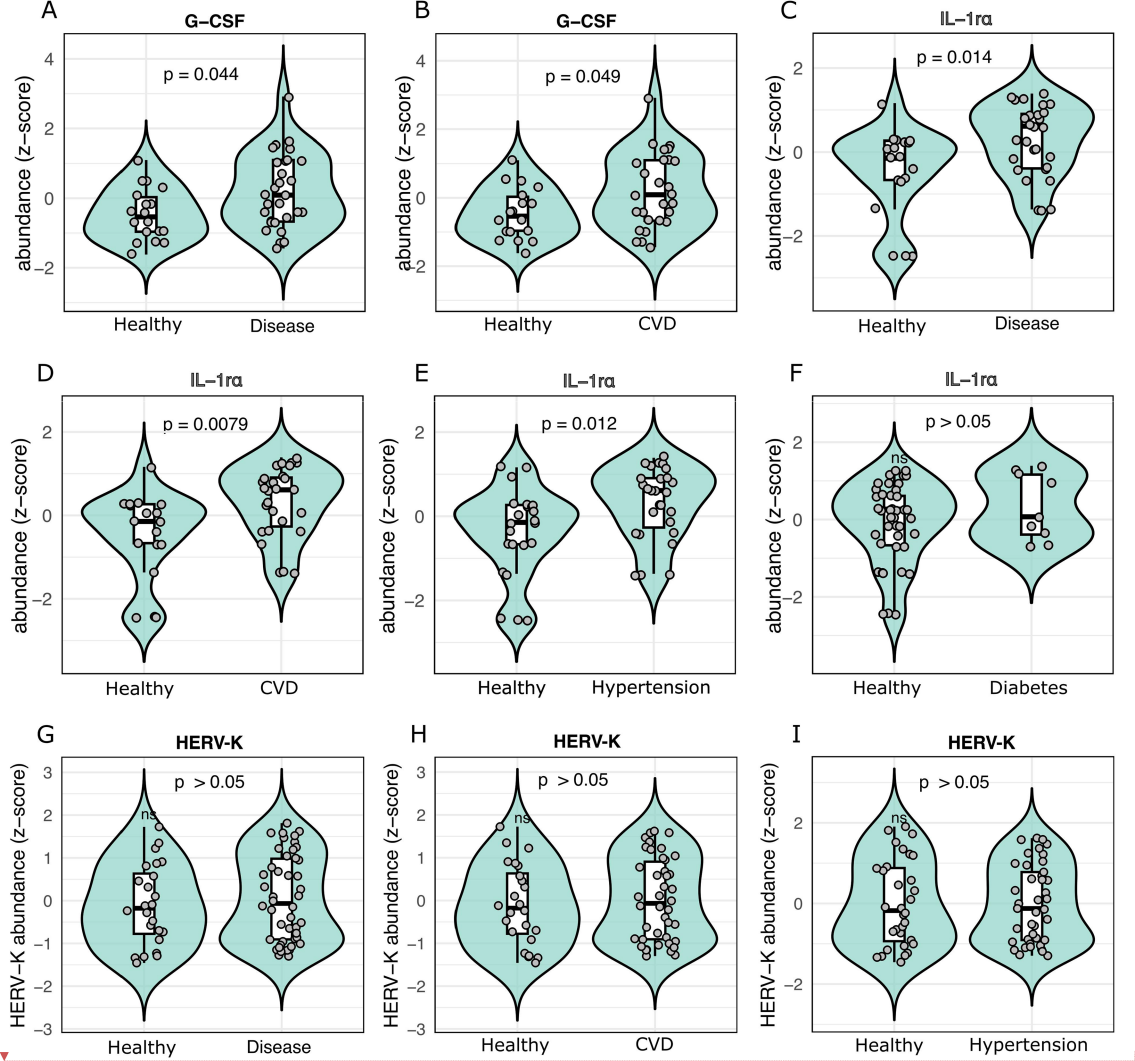
Differential abundance of HERV-K and circulating cytokines in health and disease. **(A–I)** Abundance comparison between cases and controls for G-CSF, IL-1rα, and HERV-K. For the cytokines, only those showing significant differences are displayed here.

We next profiled the abundance of HERV-K envelope protein and a panel of 27 circulating cytokines to identify systemic immune signatures associated with disease states, especially CVD, hypertension, and diabetes (Figures 5A–I). Among all analytes measured, two cytokines emerged as consistently elevated across conditions. G-CSF was elevated in overall disease status (*p* = 0.045) and CVD (*p* = 0.049) (Figures 5A,B), and IL-1r*α* was significantly higher in disease status (*p* = 0.014), CVD (*p* = 0.0075), and hypertension (*p* = 0.012), with a consistent but non-significant trend in diabetes (Figures 5C–F). Several other cytokines showed limited associations, including MIP-1a in CVD (*p* = 0.086), IL-15 in diabetes (*p* = 0.090), and VEGF in diabetes (*p* = 0.098). In contrast, HERV-K envelope protein abundance showed no significant associations, although a modest increase was observed in individuals with overall disease status and cardiovascular disease (Figures 5G–I).

### Anellovirus-cytokine correlations reveal distinct module-associated immune signatures

**Figure 6:**
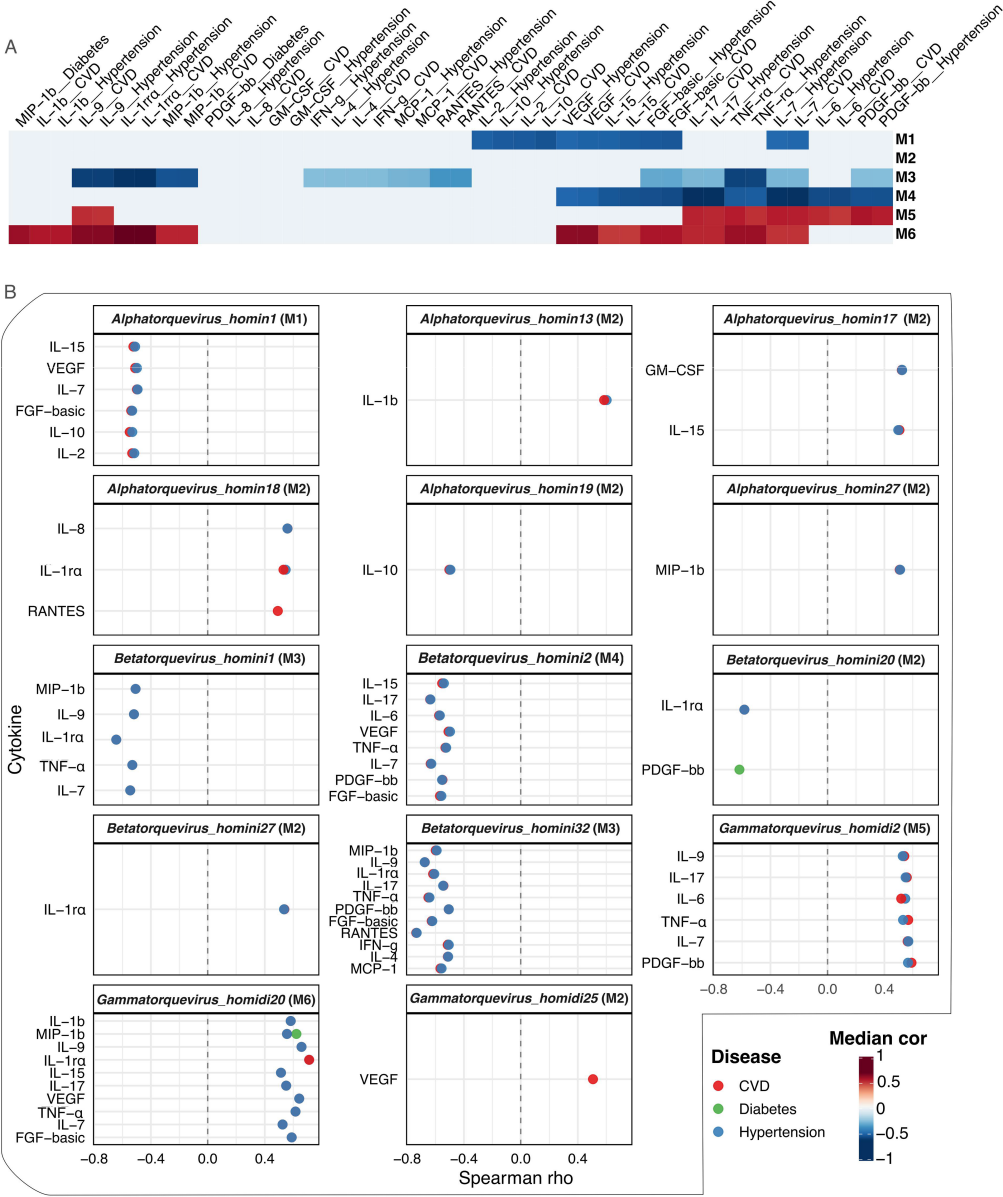
Anellovirus-cytokine correlations across clinical conditions (rho > 4). **(A)** Module-level correlation signatures. Anellovirus species modules (or clusters) were defined by hierarchical clustering of species based on their correlation profiles with cytokines across disease conditions. The heatmap displays median Spearman correlation coefficients (rho) between each virus module and cytokine-disease combination. **(B)** Species-level correlation facets. The top 12 anellovirus species with the highest number of significant cytokine associations are shown. Each panel displays Spearman correlations between the indicated virus species and cytokines, stratified by disease condition. The direction and magnitude of correlations are consistent with the module-level patterns observed in **(A)**, supporting the biological coherence of the identified clusters. Anellovirus species for each module can be found in Supplementary Table S5.

We investigated coordinated associations between anellovirus communities and systemic immune markers across clinical conditions (Figure 6, Supplementary Figure S4). Most *Alphatorquevirus* species showed weak correlations with a few negative outliers, *Betatorquevirus* species exhibited broader negative correlations with inflammatory markers, while *Gammatorquevirus* species were positively associated with diverse cytokines including IL-1rα, VEGF, and TNF-α, reflecting module-level trends across anellovirus genera (Supplementary Figure S4A).

These module-specific analyses revealed coherent patterns (Figures 6A,B, Supplementary Table S5). Module M6 and M5, dominated by *Gammatorquevirus* species (M6: *Gammatorquevirus_homidi20*; M5: *Gammatorquevirus_homidi2*), displayed strong positive correlations with systemic immune markers, indicating a reproducible pro-inflammatory and growth-factor signature. M6 showed the strongest positive median Spearman correlations, notably IL-1rα (rho ~ 0.71–0.72 for disease status, CVD, and hypertension) and IL-1β (rho ~ 0.52–0.58), plus IL-12, IL-15 and several growth factors including VEGF (rho ~ 0.49–0.66). M5 had a complementary profile with IL-17 (rho ~ 0.55–0.56 in CVD and hypertension), multiple growth factors (rho ~ 0.52–0.59), IL-6, and TNF-α.

In contrast, Modules M1, M3, and M4 displayed predominantly negative correlations (Figures 6A,B, Supplementary Figure S4B). M1 (*Alphatorquevirus_homin1*) negatively correlated with FGF-basic (rho ~ − 0.54), IL-10 (rho ~ − 0.55), and IL-2, IL-15, IL-7, and VEGF in hypertention/CVD. M3 (*Betatorquevirus_homini1* & *Betatorquevirus_homini32*) correlated negatively with IL-1rα (rho ~ − 0.63 in CVD) and other proinflammatory cytokines (RANTES, IL-9 and TNF-α, rho range: −0.4.3 to −0.63). M4 (*Betatorquevirus_homini2*) contained the strongest negative correlations, including IL-6 (rho ~ − 0.62) and IL-17 (rho ~ − 0.63) hypertention/CVD. Module M2, mostly *Alphatorquevirus* species, showed few strong cytokine correlations. Notably, some species-level correlations, including *Alphatorquevirus_homini18* with RANTES and *Gammatorquevirus_homidi25* with VEGF, were observed only in CVD. Overall, species-level patterns mirrored their module profiles, supporting the biological coherence of these immunomodulatory clusters.

## Discussion

In this study, we applied metagenomic to 218 individuals from two age-stratified cohorts to perform a high-resolution, species-level analysis of the human plasma virome and its relationship with age, disease, and systemic immunity. We found that the plasma virome is overwhelmingly dominated by Anelloviridae, with species-specific patterns tightly linked to CVD, host age, sex, and distinct immune signatures. These results provide insights into potential ecological and immunological roles of commensal viruses in human health.

Anelloviridae are ubiquitous, small, circular single-stranded DNA viruses that establish lifelong, typically non-pathogenic, bloodborne infections and comprise the dominant eukaryotic fraction of the human plasma virome. TTV is commonly used clinically as a shorthand for the Alphatorquevirus group, and most qPCR assays target conserved regions and therefore return an aggregate, genus-level signal, whereas untargeted metagenomics resolves individual species and lineages and can reveal species-specific associations that qPCR conceals.

Our results revealed pronounced age-dependent restructuring of the plasma virome. Older adults exhibited near-ubiquitous anellovirus colonization, with substantially higher abundances than young individuals, consistent with the permissive environment created by immunosenescence and inflammaging (Liu et al., 2023; Timmerman et al., 2024). Mechanistically, persistent anellovirus replication is supported by virus-encoded replication-initiation proteins that engage host DNA replication complexes and by long-term residency in leukocyte compartments (T cells and possibly granulocytes), which together plausibly enable lifelong detection and increasing abundance with age (Kosulin et al., 2018; Boisvert et al., 2025). Disease-associated shifts were pronounced at species level, with *Alphatorquevirus_homin1* and *Alphatorquevirus_homin13* markedly enriched in older diseased adults. These findings suggest that anellovirus quantification at family or genus-level may obscure critical species-level dynamics underlying disease associations. The stark contrast in machine learning classifier performance between age cohorts highlights the impact of host biology on virome-disease relatioships: high classifier accuracy in the younger cohort suggests that acute or subacute disease states produce clearer, species-specific perturbations of the anellome, whereas in older adults high baseline prevalence and accumulated host factors (multimorbidity, polypharmacy, immune remodeling) likely mask disease-specific signatures, reducing discriminatory power (Arze et al., 2021; Kaczorowska et al., 2022a; Cao et al., 2023; Kaczorowska et al., 2023; de Boer et al., 2024; Timmerman et al., 2024; Modha et al., 2025).

Prior epidemiological studies and self-controlled case-series have shown that respiratory infections, particularly influenza and SARS-CoV-2, are associated with a substantially increased short-term risk of acute AMI and ischemic stroke, as well as worse in-hospital outcomes (Warren-Gash et al., 2009; Qureshi et al., 2021; Betts et al., 2024; de Boer et al., 2024). These findings implicate inflammation-driven and prothrombotic pathways as proximate mechanisms. Our species-level anellovirus results suggest that persistent or cumulative viral exposures, or an altered host-virus equilibrium (the “infectious burden”), may act alongside acute infections to modulate longer-term cardiovascular risk.

Differential enrichment of specific anellovirus taxa in stroke and CHD, for example, increased *Samektorquevirus* and *Alphatorquevirus*/*Betatorquevirus* in stroke and reduced *Gammatorquevirus* diversity in CHD, indicates that viral taxa may engage host immunity and the vasculature through taxon-specific mechanisms (Kaczorowska et al., 2022b; Clarke et al., 2024; Modha et al., 2025). These patterns are compatible with two non-exclusive hypotheses: (i) certain commensal viruses occupy immunological niches that promote low-grade inflammation and vascular vulnerability, thereby contributing to disease pathogenesis, or (ii) disease processes, clinical therapies, or altered immune control selectively permit expansion of particular taxa (Zheng et al., 2020; Hou et al., 2022).

Detection of low-prevalence eukaryotic viruses almost exclusively in older adults may reflect cumulative lifetime exposure, episodic reactivation, or reduced clearance. Plasma detection, however, does not establish active replication or tissue tropism and should be confirmed by targeted longitudinal sampling or tissue assays. Although these taxa were rare and non-predictive in our models, their age-restricted presence suggests they may serve as ecological markers of virome ageing and therefore warrant longitudinal follow-up, particularly given evidence that acute viral infections can produce persistent cardiometabolic sequelae such as new-onset diabetes and hypertension (Birabaharan et al., 2022; Miller et al., 2024; Boparai et al., 2025; Kanouse et al., 2025). Additionally, the pronounced male bias in anellovirus prevalence, coupled with only modest associations for lifestyle factors, suggests that intrinsic biological differences, such as sex-linked immune or hormonal regulation, are likely stronger drivers of the anellome than behavioral exposures in this older adult cohort.

Sex-dependent remodeling of the anellome in older adults aligns with established sex differences in immune aging and hormone-mediated immune modulation (Huang et al., 2021). The sensitivity of anellovirus load to host immune competence, documented in previous studies, further supports that sex-specific immune trajectories will shape anellome composition (Kyathanahalli et al., 2021; Thijssen et al., 2023; Cebriá-Mendoza et al., 2023). Our observation that aged males display higher *Alpha*- and *Gammatorquevirus* species diversity while older females show relatively greater *Samektorquevirus* species diversity aligns with these expectations and underscores the need to stratify virome analyses by both sex and age.

Across disease states, IL-1r*α* and G-CSF, were consistently elevated, particularly in CVD, reflects innate immune activation and compensatory regulation. IL-1rα, a natural IL-1β antagonist, is linked to vascular inflammation and cardiometabolic risk in aging populations (Almeida-Santiago et al., 2022; Yang et al., 2023; Wang et al., 2025). Mendelian randomization analyses indicate that genetically elevated IL-1rα increases coronary heart disease and myocardial infarction risk, largely via apolipoprotein B-related lipid pathways (Yang et al., 2023). Lifestyle interventions that reduce IL-1rα may therefore mitigate cardiovascular risk. In parallel, G-CSF, a key mediator of neutrophil dynamics and tissue repair, is implicated in cardiovascular inflammation and angiogenesis (Mulazzani et al., 2023). Together, these cytokine shifts offer insights into host immune status and vascular vulnerability, suggesting potential biomarkers for risk stratification.

A recent study showed that anelloviruses exploit host replication machinery through ORF2/3-mediated initiation and a recombination-dependent mechanism, enabling persistent infection (Boisvert et al., 2025). Structural analyses revealed anellovirus icosahedral capsids with hypervariable surface spikes that conceal conserved domains and present diverse epitopes, supporting immune evasion and species-specific host footprints (Liou et al., 2024). Exposure to TTV DNA has been shown to activate TLR9 and induce pro-inflammatory cytokines in ex-vivo and cell-based assays, but direct evidence that active anellovirus replication triggers TLR9 *in vivo* is currently lacking; thus, the relevance of TLR9 signalling for persistent anellovirus infections requires further study (Rocchi et al., 2009). These molecular features, combined with leukocyte residency, help explain why species-level differences exhibit and how they may exhibit distinct immunological signatures (Gore et al., 2023).

Integration of virome and cytokine data provides mechanistic context for taxon-specific associations. *Gammatorquevirus*-dominated modules and some *Alphatorquevirus* species correlated positively with IL-1 pathway components, TNF-α and multiple growth factors, consistent with NLRP3/IL-1-driven innate activation, Th1/Th17 polarization, endothelial activation, and acute-phase responses implicated in vascular inflammation and cardiometabolic disease (Toldo and Abbate, 2018; Li et al., 2024; Clarke et al., 2024; Nguyen et al., 2025; Zhou and Feng, 2025). In contrast, *Betatorquevirus*-dominated modules inversely correlated with several inflammatory mediators including RANTES, IL-9 and TNF-α, suggesting a more tolerogenic or regulatory circulating niche (Gore et al., 2023; Zhang et al., 2023). Notably, *Alphatorquevirus_homin1* negatively correlated with IL-2, IL-15, IL-7, VEGF and IL-10. Both IL-10 and VEGF are key mediators in cardiovascular and metabolic health: IL-10 protects against atherosclerosis and improves insulin sensitivity, while VEGF can support ischemic repair but may exacerbate diabetic microvascular complication. These observations support a model in which some anellovirus species adopting different host-interaction strategies, with some aligning with pro-inflammatory networks relevant to cardiovascular pathology, others occupying regulatory niches, and many showing weak or inconsistent immune correlations (Liou et al., 2024).

Causality and directionality remain unresolved. Acute infections can provoke cardiovascular events via inflammatory and thrombotic mechanisms, while persistent viruses may reset immune set-points and modulate long-term vascular risk. Our data accommodate both hypotheses: specific anellovirus species might contribute to inflammatory milieus that increase cardiometabolic risk or simply reflecting immune remodeling. We recommend prospective studies that pair standardized TTV qPCR (for longitudinal immune surveillance) with species-level metagenomics and targeted functional assays to validate candidate species as mechanistic biomarkers.

Our analysis found no significant difference in serum HERV-K envelope protein levels between individuals with disease or cardiometabolic conditions and healthy controls. This suggests that circulating HERV-K envelope protein may not be a robust biomarker for these conditions in our cohort. This result contrasts with studies linking other HERV elements to related diseases, such as HERV-K dUTPase in pulmonary hypertension or HERV-H/W transcripts in type 1 diabetes (Saito et al., 2017; Tovo et al., 2020). These discrepancies likely reflect fundamental biological differences, including the specific HERV family, the targeted protein (envelope vs. dUTPase), and the compartment measured.

This study has several limitations that warrant consideration. Although our cohort (*n* = 218) enabled broad virome characterization, statistical power was limited for some diseases including CHD and Stroke. Using REPLI-g WTA kit which is based on Phi29 amplification likely biased detection toward small circular DNA viruses. Moreover, the aggregation of chronic diseases in the young cohort precluded disease-specific interpretations. The investigation of anellovirus diversity in relation to clinical traits was constrained in the young cohort by the small number of diseased children, and in the older cohort by the small sample sizes resulting from stratification by both sex and specific diseases. The identified associations for specific anellovirus species require validation in an independent cohort. The cross-sectional design prevents causal inference, and plasma metagenomics may miss tissue-specific or extremely low-abundance viruses. Because anelloviruses can persist in leukocyte compartments and study of their biology has been limited by historically poor culture systems, plasma metagenomics may under-represent tissue-resident dynamics. Unexplored confounders including medication or vaccination history, suggest the need for cautious interpretation. Moreover, differences in early-life environments across the decades may explain the differences of anellovirus findings between age groups. Additionally, we did not measure CMV serostatus. Since CMV is more prevalent in older adults, and being positive for CMV is associated with higher anellovirus concentrations, CMV may be a confounding factor in our age-associations (Haloschan et al., 2014).

Despite these constraints, species-resolved virome profiling reveals meaningful associations between anellovirus composition, host immunity, and cardiometabolic traits. Species-level shifts in abundance, diversity, and immune correlates, highlight the limitations of family- or genus-level quantification and emphasize the value of high-resolution viromics. Future studies should prioritize larger, multi-center cohorts with longitudinal sampling, broader age ranges (including mid-life adults), and richer clinical metadata to clarify directionality, elucidate mechanisms of virome–host interactions, and assess the translational potential of key anellovirus species as biomarkers of immune function or disease risk.

## Data Availability

The metagenomic data generated in this study were deposited in the Sequence Read Archive under the BioProject number PRJNA1314973. The datasets presented in this study can be found in online repositories. The names of the repository/repositories and accession number(s) can be found in the article/Supplementary material.

## Footnotes

1
https://github.com/Laubscher/Anelloviruses/releases/tag/Anellovirus_2025.2

## Supplementary material

FIGURE S1. Overview of the study workflow and virome bioinformatics. Workflow of the study including viral metagenomic, cytokines and HERV-K measurement assys **(A)**. Viral bioinformatic from raw read preproceesing to taxonomic anotation **(B)**.

FIGURE S2. Metagenomic overview of the study cohorts. Total metagenomic **(A)** and viral hit contigs by cohort. Quality tier distribution by age bin in young **(C)** and old **(D)** cohorts. Complete viral contigs by cohort and disease status **(E)**. Viral species exclusively found in old cohort **(F)**.

FIGURE S3. Plasma virome alpha diversity in healthy individuals by age cohort and sex. Only results with corrected *P*-values < 0.1 are shown for explorative signal. Shannon diversity of viral families by cohort **(A)** and Richness of Anellovirus genera by sex in the old cohort **(B)** Shannon diversity of Alphatorquevirus species by cohort **(C)**. Richness of Alphatorquevirus species by cohot **(D)**. Shannon diversity of Alphatorquevirus species by sex in the old cohot. Richness of Alphatorquevirus species by sex in old cohort. Shannon diversity of Gammatorquevirus species by sex in the old cohort **(G)**. Richness of Gammatorquevirus species by sex in the old cohort **(H)**. Shannon diversity of Samektorquevirus species by sex in old cohort **(I)**.

FIGURE S4. Top anellovirus species-cytokine correlations across each genus **(A)**. Cytokines negatively associated with *Alphatorquevirus_homini1* with the top 6 highlighted in red **(B)**.

TABLE S1. Patients’ metadata.

TABLE S2. Metagenomic assembly.

TABLE S3. Predictive modeling of anellovirus species for diseas-status vs health across cohorts.

TABLE S4-10. Viral read abundance data across viral taxa and cytokine and HERV-K measurements.

TABLE S4. Logistic regression of anelloviruses prevalence across age, sex, and lifestyle factors.

TABLE S5. Module species and residualized correlation data.

TABLE S6. Viral read abundance data across viral taxa and cytokine and HERV-K.
